# Cloudy with a Chance of Insights: Context Dependent Gene Regulation and Implications for Evolutionary Studies

**DOI:** 10.3390/genes10070492

**Published:** 2019-06-28

**Authors:** Elisa Buchberger, Micael Reis, Ting-Hsuan Lu, Nico Posnien

**Affiliations:** 1 Dpt. of Developmental Biology, Göttingen Center for Molecular Biosciences (GZMB), University Göttingen, Justus-von-Liebig-Weg 11, 37077 Göttingen, Germany; 2 International Max Planck Research School for Genome Science, Am Fassberg 11, 37077 Göttingen, Germany

**Keywords:** gene expression, gene regulation, evolution, allele specific expression, expression quantitative trait loci (eQTL), RNAseq, ChIPseq, chromatin, Assay for Transposase-Accessible Chromatin using sequencing (ATACseq), genotype–phenotype map

## Abstract

Research in various fields of evolutionary biology has shown that divergence in gene expression is a key driver for phenotypic evolution. An exceptional contribution of *cis*-regulatory divergence has been found to contribute to morphological diversification. In the light of these findings, the analysis of genome-wide expression data has become one of the central tools to link genotype and phenotype information on a more mechanistic level. However, in many studies, especially if general conclusions are drawn from such data, a key feature of gene regulation is often neglected. With our article, we want to raise awareness that gene regulation and thus gene expression is highly context dependent. Genes show tissue- and stage-specific expression. We argue that the regulatory context must be considered in comparative expression studies.

## 1. Introduction

**Figure 1:**
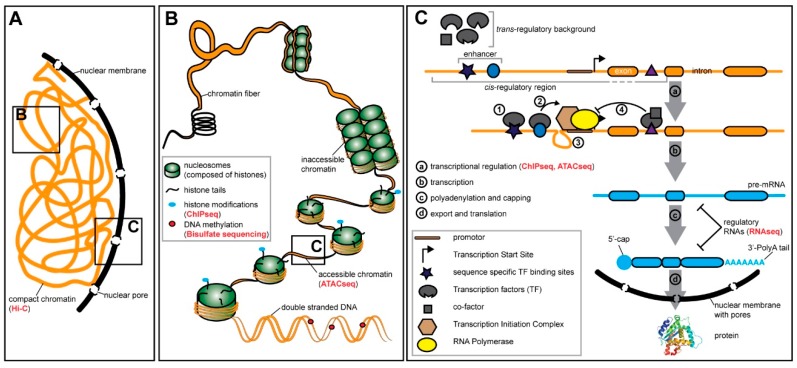
Gene expression is regulated on various levels. (**A**) The DNA is compressed in the nucleus of the cell. (**B**) The DNA in the nucleus is compressed by binding of histone proteins. The chromatin contains easily accessible euchromatin regions and highly compact and inaccessible heterochromatin regions. The status of the chromatin is influenced by post-translational histone modifications. Gene expression is modulated by the chromatin state and DNA modifications, such as methylations. (**C**) Key steps of gene expression (**a**–**d**). Transcription factors (TFs) bind to the DNA at specific sequences (**1**). TF binding activates the transcription initiation complex (**2**) through conformation changes (looping) of the DNA (**3**). TFs can also repress transcription, for instance by binding of a co-factor (**4**). Next generation sequencing (NGS)-based methods that can be applied to study certain aspects of gene regulation are mentioned in red in brackets. See Table 1 for an overview of the methods mentioned here.

Living organisms are uniquely characterized by their appearance, their function, and their interaction with the environment. The information about these features is provided in the genome which is packed into the nucleus of each cell (see Figure 1A). Various disciplines of biological and medical research aim at understanding how the genomic information is transformed into organismic functionality. Proteins and peptides are the molecules that accomplish manifold tasks in an organism, such as orchestrating its development [1], providing energy through metabolism [2,3], protection via immune responses [4,5], and processing environmental information in the nervous system [6,7]. Protein and peptide sequences are encoded in gene regions of the genome. Genes are transcribed into ribonucleic acid (RNA) molecules that serve as templates for the translation machinery that eventually synthesizes functional proteins. This process, called gene expression, is thus crucial for every living organism. 

Since the identification of deoxyribonucleic acid (DNA) as genetic material in 1944 [8] a major focus in Evolutionary Biology and Quantitative Genetics has been to reveal the connections between variation in DNA sequences and phenotypic differences observed among organisms (i.e., the genotype–phenotype map) [9,10,11,12]. If causative genetic variation is identified in protein coding sequences it is straightforward to directly link these differences to changes in protein function [13,14,15,16]. However, if causative genetic variation is present in intergenic or intronic (i.e., non-coding) sequences it is less intuitive to infer direct links between the observed variation and phenotypic differences. Since these non-coding regions may be important regulatory sequences it is conceivable to connect genetic variation in such regions with differential gene expression. With the advent of efficient and affordable sequencing technologies (next generation sequencing, NGS) it became feasible to study gene expression on a genome wide scale [17]. Since these technologies also provide the opportunity to obtain such data in plant and animal systems beyond well-established genetic models, gene expression has extensively been used as proxy for genetic variation to gain insights into phenotypic evolution [18,19].

In this review we will first summarize findings illustrating the importance of gene expression divergence in phenotypic evolution for various morphological, behavioral, physiological, and life-history traits. Next, we will present current approaches aiming at understanding genome-wide patterns of gene expression divergence as well as the underlying molecular mechanisms. We will review various mechanisms underlying gene regulation and we will highlight how they facilitate context dependent gene expression. We argue that gene expression and gene regulation evolve in a highly context dependent manner and we will suggest to consider that knowledge to improve the efficiency of comparative gene expression studies.

## 2. Gene Expression Divergence Affects Phenotypic Evolution

Changes in gene expression have been linked to variation in many phenotypes. In the last years, there has been an increase in the number of ecological and evolutionary studies using transcriptomics to understand how environment and different life strategies affect gene expression [20,21]. Most examples found in the literature connecting genetic variation affecting gene expression with phenotypes are based on studying simple morphological traits, such as the evolution of trichome patterns in *Drosophila* or differences in body coloration. For instance, a clear link between changes in the regulatory region of the *shavenbaby* gene and the evolution of trichome patterns across *Drosophila* species has been established [22,23]. Similarly, individual nucleotide polymorphisms in the *ebony* [24] and *yellow* genes [25] underlie natural differences in body and wing pigmentation, respectively, in *Drosophila*. Divergence in fur coloration in mice has been shown to be regulated by differences in developmental expression of the gene *agouti* [26,27]. Moreover, the stripe pattern of cichlid fishes is associated with differential expression of the gene *agouti-related peptide 2* (*agrp2*) [28]. 

Besides these classical traits, also more complex traits are being studied. In *Drosophila*, the shape of male genitalia evolves rapidly, contributing to speciation processes. Divergence in the expression of the *tartan* gene has recently been shown to contribute to interspecific differences between *D. mauritiana* and *D. simulans* [29]. Another study has shown that a single nucleotide change in the *cis*-regulatory region of *scute* has pleiotropic effects by affecting genitalia bristle and sex comb sensory teeth number simultaneously [30]. Hence, gene expression divergence is a major driver of the evolution of morphological traits.

Recently it has been argued that the molecular architecture of differences in behavioral traits may be simpler than previously anticipated. For instance, a complex behavior such as sociality in bees has been shown to be clearly associated with differential expression of the gene *syntaxin1a*, since higher expression of this gene is directly correlated with a social life style [31]. Similarly, differences in parental care between the promiscuous deer mouse (*Peromyscus maniculatus bairdii*) and its sister species, the monogamous old-field mouse (*P. polionotus subgriseus*) is influenced by differential expression of the gene *vasopressin* [32]. These examples impressively demonstrate that the evolution of behavioral traits is associated with divergence in gene expression.

Many studies exploring the molecular basis of the evolution of physiological and life-history traits followed by functional validation have confirmed an underlying polygenic architecture [12,33,34]. Nevertheless, few studies reached the resolution to narrow down genetic variation to the level of individual loci. A recent study in European aspen (*Populus tremula*) has shown that expression divergence of a single gene (*PtFT2*) is responsible for 65% of the differences in timing of bud set [35]. Other studies similarly identified mutations in *cis*-regulatory regions causing gene expression divergence which ultimately affects an organism’s physiological response to the environment. For example, a 2 bp deletion in the promoter region of the gene *ERG28* in *Saccharomyces cerevisiae* results in reduced expression associated with resistance to an antifungal drug [36]. Similarly, an indel in the 3’UTR of *MtnA* that shows signatures of selection, causes a 4-fold difference in gene expression and confers resistance to oxidative stress in natural populations of *D. melanogaster* [37].

In summary, genetic variation associated with the evolution of phenotypic traits such as morphology, behaviour, life history, and physiology often affect gene expression. Therefore, gene expression divergence is a major driver for phenotypic evolution.

## 3. Gene Expression Divergence Reflects Divergent Gene Regulatory Mechanisms

Since gene expression divergence is often linked to phenotypic evolution, many comparative studies employ gene expression as intermediate phenotype to link genetic variation to trait divergence. However, besides using gene expression to establish genotype–phenotype maps, the availability of high throughput methods to survey genome wide expression levels also allows to study global patterns of gene expression divergence. It has for instance been shown that divergence in gene expression is pervasive among populations in *Drosophila* [38], yeast [39], or in fish [40]. It is therefore likely that genetic variants affecting gene expression segregate in natural populations and can be selected for [41]. Also, interspecific gene expression data across seven *Drosophila* species has been used in modelling approaches integrating fitness estimates and a phylogenetic framework to reveal that expression divergence shows signatures of directional selection [42]. Additionally, interspecific gene expression comparisons contributed to a better understanding of biological phenomena such as sex-biased gene expression [43] or expression variation of duplicated genes [44]. Therefore, comparative gene expression studies revealed a high level of variation in gene expression within and among species.

The accumulation of comparative genome wide expression data triggered a strong interest in unravelling the molecular and evolutionary mechanisms underlying divergence in gene expression itself. Most of our current mechanistic understanding of gene expression divergence is based on work in genetic model systems that are tractable for genetic crosses. Two main methods have been employed extensively in recent years, i.e., expression quantitative trait loci (eQTL) mapping and allele-specific expression studies (ASE). eQTL studies are basically QTL or genome wide association (GWAS) studies aiming at identifying causative loci responsible for gene expression variation. Conceptionally, this method assumes that the level of gene expression can be treated as a quantitative trait [45,46,47]. Therefore, normal QTL or association mapping methodology can be applied to reveal genomic variants associated with expression divergence. eQTL studies supported the fundamental observation that gene expression is indeed highly variable across individuals and heritability estimates support the contribution of a genetic component [48].

While eQTL studies reveal genomic loci or individual single nucleotide polymorphisms (SNPs) associated with expression difference, ASE studies in F1 hybrids represent a powerful approach to gain mechanistic insights into differential gene expression [49]. The analysis of gene expression between homozygous parents (closely related species or populations of the same species) and the allele specific expression in their heterozygous F1 offspring allows distinguishing whether a gene is differentially expressed due to changes in its own regulatory region (*cis*-regulatory divergence) or due to changes somewhere else in the genome (*trans*-regulatory divergence) [50,51]. *cis*-regulatory divergence is inferred if two different alleles of a given gene have a major impact on its allele-specific expression in the homogenous *trans*-regulatory background of the F1 hybrid. *trans*-regulatory divergence is inferred if a gene is differentially expressed between two parental individuals, but the contribution of the two alleles in the hybrid background is the same. The most consistent observation in ASE studies is that *cis*-regulatory divergence seems to be prevalent in intra- as well as interspecific comparisons [52,53,54,55]. Exceptions have been observed for instance for comparisons between the cosmopolitan fly species *D. melanogaster* and the closely related specialist species *D. sechellia* [56]. In all mentioned ASE studies, a major impact of a combination of *cis*- and *trans*-divergence has been observed, strongly supporting the notion that gene regulation is complex and thus can evolve in complex patterns. 

ASE studies alone do not allow revealing genetic variants associated with gene expression divergence. However, one of the most likely explanation for *cis*-divergence effects is sequence variation in the regulatory region (i.e., promoters or enhancers) of the differentially expressed gene. Indeed, in putative regulatory regions of genes showing *cis*-regulatory divergence increased levels of sequence divergence have been found in yeast [57], *Arabidopsis thaliana* [58], maize [59], and *Drosophila* [56,60]. A combination of ASE and SNP data obtained from lymphoblastoid cell lines from the 1000 Genomes Project further strongly suggests that genetic variation is a common explanation for allele-specific gene expression [61]. Hence, the combination of ASE and eQTL studies provide exceptional insights into gene expression divergence. 

In summary, comparative gene expression studies are extensively used to establish genotype–phenotype relationships and to reveal global patterns of expression variation. eQTL and ASE furthermore represent excellent approaches to gain mechanistic insights into gene expression divergence.

## 4. Gene Expression and Gene Regulation are Highly Context Dependent

Many of the above-mentioned exciting fundamental insights into gene expression divergence are based on studies in entire organisms and adult stages. However, it is broadly accepted that gene expression is strongly context dependent with a major impact for instance of the developmental stage and the tissue [62]. Focusing comparative gene expression studies on a few stages of an organism’s life history and a combined view of usually complex tissue compositions underestimates this important aspect of gene expression and gene regulation. Since the molecular mechanisms underlying context dependent gene regulation are being revealed these days, we will summarize key features of the gene regulation machinery and highlight how they facilitate context dependent gene expression. 

### 4.1. Pre-transcriptional Regulation—Chromatin States and Methylation

The first regulatory mechanisms are at play on the level of genome organisation. Compressed DNA in the nucleus forms a tertiary structure (Figure 1A) that can be studied in detail by an NGS based chromosome conformation capture method called Hi-C [63] (Table 1). Hi-C applied in various bilaterians revealed one fundamental characteristic of the genome: Some regions of the genome interact consistently more often than other regions [64,65,66]. These topologically associating domains (TADs) have been shown to influence gene expression. For instance, the famous temporal and spatial collinearity of *Hox* gene expression in the developing vertebrate limb has been associated with the location of the *HoxD* cluster in a gene desert that lies between two adjacent TADs [67]. The application of Hi-C in different human primary blood cell types showed that these interactions are highly cell-type specific [68]. How the three-dimensional organization of the genome exactly influences gene regulation has just started to be revealed and represents an active and exciting field of research.

Highly transcribed regions of the genome (i.e., euchromatin) are—in contrast to the condensed heterochromatin—usually depleted of nucleosomes (Figure 1B). These nucleosome free regions can be detected on a genome wide scale using NGS based methods such as ATACseq (Assay for Transposase-Accessible Chromatin using sequencing) [69] (Table 1). Recent application of ATACseq on single cells originating from 13 different mouse tissues [70] and from three stages of *Drosophila* embryonic development [71] revealed clear signatures of cell type and stage specific chromatin accessibility states. The chromatin state and thus DNA accessibility is influenced by cell type specific modifications of histone proteins [72,73], the subunits of nucleosomes, and is clearly linked to gene regulation [74,75]. Even if DNA is accessible, the transcription of genes can be modulated by DNA methylation, i.e., the addition of a methyl group to cytosines. DNA methylation has been associated with gene repression [76,77] and recent data has shown that transcription factors can integrate methylation patterns to refine gene regulation [78]. Since the methylation is highly dynamic, for instance throughout cellular differentiation [79], it facilitates context dependent gene regulation. 

Chromatin accessibility and genome architecture is also regulated by a variety of non-coding RNA molecules, which are transcribed, but not translated into proteins. Long non-coding RNAs (lncRNAs) localized in the nucleus can directly affect chromatin architecture [80]. Intriguingly, lncRNA–protein interactions are tissue specific since they were observed in mouse placenta cells, but not in liver tissue [81]. lncRNAs have also been implicated in transcriptional activation since they mediate active histone marks [82] and stabilize enhancer-promotor interactions [83]. Micro RNAs (miRNAs), another group of non-coding RNAs, have been shown to directly modulate histone modifications and thus the chromatin accessibility to allow transcription of target genes [84]. Since miRNAs are highly tissue specifically expressed [85,86,87], these molecules provide an excellent mechanism to facilitate tissue specific chromatin accessibility.

In summary, the extensive diversity of epigenetic modifications, which are further modulated by non-coding RNAs regulate differential DNA accessibility and thus provide a rich cellular repertoire to control gene expression pre-transcriptionally.

### 4.2. Transcriptional Regulation—Transcription Factors and Cis-Regulatory Elements

Once the chromatin is accessible for proteins, gene expression is directly regulated by protein–DNA interactions [88,89] (Figure 1C). Transcription factors are proteins with dedicated DNA-binding domains and their sequence specific binding fosters or represses gene expression. A classic example for context-specific gene regulation via transcription factors is the development of different neuronal subtypes in the *Drosophila* central nervous system. Initially, all neuronal precursor cells, the neuroblasts, contain generic neuronal transcription factors [90]. The unique identity of each neuroblast is further specified by spatial and temporal cues. Different neuronal subtypes are defined by the expression of temporally restricted transcription factors [91] and the regional identity of neuroblasts is regulated by the expression of spatially restricted transcription factors [92]. Therefore, context dependent gene regulation can be achieved on the level of the presence of transcription factors which are expressed cell and time specifically. 

Besides the transcription factors itself, the nature of the DNA sequences they bind to plays a major role in gene regulation. These *cis*-regulatory regions can be subdivided based on their location relative to the respective gene locus (Figure 1C). Promoters lie directly upstream of the transcription start site and general transcription factors bind there as part of the transcription initiation complex [93,94]. Enhancers are *cis*-regulatory sequences that are located further away up- and downstream of the transcription start site. They are composed of distinct sequence motifs that are specifically recognized by certain transcription factors. Transcription factors bound to enhancers facilitate the assembly and activation of the transcription initiation complex at the promoter [95,96]. Although we focus here on enhancers, many of the discussed aspects apply to other elements, such as silencers and insulators as well. Enhancers are highly modular [97,98], as exemplified by the regulation of the pair-rule gene *even skipped (eve)* during segmentation in the *Drosophila* embryo. The seven stripes of *eve* expression are spatially defined by five enhancers with each of them being responsible for an individual stripe or a pair of stripes [99,100]. Therefore, the modular nature of enhancers provides a source for context-dependent activation (and repression) of genes. 

The interaction of transcription factors and *cis*-regulatory elements can be further diversified by the interaction of transcription factors with co-factors that are expressed in a temporally and spatially defined manner to modulate for instance their capacity to bind to regulatory regions (Figure 1C). One excellent example for the context dependence of gene regulation achieved via the spatial availability of co-factors has been shown in the developing wing disc of *Drosophila*. During wing development, the transcription factor Pannier (Pnr) can act as an activator in some regions, while the presence and binding of its co-factor U-shaped (Ush) transforms it into a transcriptional repressor in adjacent regions [101,102,103]. The importance of transcriptional co-factors has also been shown on a genome wide scale. For instance, the two transcription factors CLOCK (CLK) and CYCLE (CYC), which are core components of the circadian clock in flies, are broadly expressed. However, the tissue specific response to the circadian clock is defined by the action of co-factors, which modulate the DNA binding capacities of these two transcription factors in a tissue specific manner [104]. The modulation of protein-DNA interactions by co-factors bound to transcription factors thus provides an additional mechanism to ascertain context-dependent gene expression.

In summary, the interaction of spatially and temporarily expressed transcription factors, with modular regulatory DNA sequences specifies the unique transcriptional landscape of a developing cell or cell groups. 

### 4.3. Post-transcriptional Regulation—RNA Modifications and Regulatory RNA Molecules

Apart from the regulation of transcription itself, the transcriptional outcome can be fine-tuned on the level of the messenger RNA. For instance, post-transcriptional modifications, such as polyadenylation and capping influence mRNA export, stability and translation efficiency [105,106,107]. Differential splicing of primary transcripts allows enlarging the repertoire of proteins to be translated from a limited number of primary RNAs. Splicing is mediated by a specific protein-complex [108] and it has been shown that tissue and cell type specific patterns of splicing factor expression recapitulate the extent of alternative spliced transcripts present in the respective tissue [109]. 

Post-transcriptional gene regulation is also mediated by regulatory RNA molecules, which can be involved in negative gene regulation via the RNA interference (RNAi) pathway (e.g., miRNA) [110] or they are part of RNA–protein complexes (e.g., lncRNA) where they influence gene regulation on various levels [111]. lncRNAs present in the cytoplasm also influence mRNA stability [112,113] and they can protect mRNA against targeted degradation by trapping miRNAs in a sponge-like mechanisms [114]. Regulatory RNAs play a major role during development [111,115] and their expression has been shown to be cell type specific [116,117]. Context dependent gene regulation can thus be mediated post-transcriptionally by differences in generic RNA modification programs (e.g., splicing) or by the action of regulatory RNA molecules (miRNA, lncRNA).

## 5. The Evolution of Gene Expression and Gene Regulation is Context Dependent

So far, we established that variation in gene expression is pervasive within and across species and that it is a major driver of phenotypic divergence. Furthermore, we showed that the different levels of the gene regulation machinery facilitate context dependent gene expression. However, few comparative expression studies have specifically tested for the context dependency of gene expression variation across species. A study of six homologous organs in nine mammals and one bird, for instance, showed that gene expression evolves at different speeds in different tissues as well as in different lineages. While gene expression was stable in the nervous system, it evolved more rapidly in testes. Similarly, gene expression variation was less pronounced in rodents compared to apes [149]. Comparative studies have also been employed to assess the impact of developmental stages on the evolution of gene expression. The analysis of expression data from various developmental stages in different vertebrates revealed the pharyngula stage to be most constraint (i.e., most similar) [150,151]. Intriguingly, a similar analysis restricted to the developing brain, instead of entire embryos, identified a stage of high conservation of gene expression much later just before birth [152]. These examples clearly demonstrate that the gene expression context, such as the type of tissue or the developmental stage, poses constraints on the overall evolvability of gene expression. Thus, the context in which gene expression variation is studied will dramatically affect the results.

A comprehensive understanding of the regulatory mechanisms underlying the context dependent evolution of gene expression is still missing to date. Indeed, few eQTL and ASE studies specifically compared findings across different tissues or stages to reveal context dependent regulatory mechanisms. A comparison between mouse embryonic and adult tissue has shown that many more distal (“*trans*”) eQTLs were found in adults compared to the investigated embryonic stage [153]. Similarly, the analysis of sexually dimorphic gene expression in different organs in intercrosses of two inbred mouse strains revealed tissue specific eQTL regions, suggesting that expression differences between sexes are regulated by tissue specific regulatory elements [154]. ASE studies have revealed different contributions of *cis*- and *trans*-divergence (or combinations thereof) by comparing differently aged flies [155] and when data from entire fly bodies was compared to heads only [54,56]. Moreover, a recent ASE study using tissue specific data for Malpighian tubules of different *D. melanogaster* populations further supports the need for more defined analyses [156]. In the light of context dependent gene regulation these first results call for an integration of stage or tissue specific aspects of gene expression in eQTL and ASE studies in order to reveal whether patterns observed so far will hold true across highly variable regulatory environments.

Indeed, the combination of various genome wide datasets for a highly context specific cellular system has already contributed to exciting insights into the impact of natural genetic variation on the different levels of the gene regulation machinery (see Figure 1). As part of the HapMap2 [157] and 1000 Genomes Project [61] lymphoblastoid cell lines were established from hundreds of individuals and subjected to genome sequencing, providing a solid basis for association studies for various regulatory traits in combination with expression variation. These studies revealed SNPs affecting all levels of gene regulation including genome organization [158], chromatin accessibility [159], histone modifications, RNA-Polymerase II occupancy, and eventually gene expression [160,161]. About 65% of the eQTLs (i.e., variation in gene expression) are associated with histone modifications and chromatin accessibility [162], suggesting that additional mechanisms must contribute to gene expression divergence. Post-transcriptional processes such as mRNA splicing are excellent candidates since individual SNPs have been associated with differences in splicing [162,163]. Since the spliceosome is already assembled during ongoing transcription, the chromatin state and the transcription rate can influence splicing events [164]. Interestingly, many identified SNPs affect different regulatory mechanisms simultaneously. For instance, genetic variants that confer higher transcription factor binding affinity are also associated with an increase in active histone marks [160], suggesting a tight causal link between transcription factor binding and histone modifications. Another link has been established between natural variation in epigenetic methylation patterns and gene expression, by showing that the same genetic variant is associated with variation in gene expression and the methylation of a CpG island close to the respective gene locus [165]. Variation in gene expression, gene regulation and methylation are therefore tightly linked.

In the light of recent findings that the rate of gain and loss of active enhancer elements in five closely related *Drosophila* species is relatively high [166], it is conceivable that natural genetic variation very quickly affects gene expression on various levels ranging from transcription factor binding to histone modification and chromatin accessibility. Additionally, a comparative study of methylation in promotor regions of primates has shown that methylated CpG islands are characterized by a higher mutation rate and that the loss of CpG islands in humans is most likely driven by methylation in sperm [167]. The observation that many regulatory traits are functionally linked and thus similarly affected by the same SNPs may explain why natural variation in gene expression is pervasive and can drive phenotypic diversification. Since many of the genome wide sequencing methods (Table 1) are readily applicable in various systems, more studies in the future will allow establishing clear links between natural variation on various levels of gene regulation and tissue-, cell-, and stage-specific evolution of gene expression. 

Since the complexity of regulatory interactions is highly context dependent, tissue-, stage-, or species-specific constraints may also be imposed by the gene regulatory networks in which certain gene products act. In the yeast *Saccharomyces cerevisiae* for instance, endogenous processes like cell cycle progression are regulated by highly complex networks, while simpler networks regulate processes that result from external stimuli (e.g., stress response) [168,169]. Highly connected genes in more complex gene regulatory networks tend to be more conserved within and across *Drosophila* species [170]. Similarly, gene regulatory networks are extensively rewired throughout development. For instance, the gene regulatory network underlying *Drosophila* trichome formation at larval and adult stages, respectively, is fundamentally different [171]. Accordingly, genetic variants affecting the expression of different genes have been identified to drive evolution in trichome patterns at different developmental stages [172,173]. Highly pleiotropic genes are important for different processes and thus they are involved in many gene regulatory networks. Therefore, the level of pleiotropy of a gene may also have an impact on its evolvability. Indeed, the expression of genes that are expressed in many tissues, i.e., pleiotropic factors, is more often conserved across species compared to tissue specific genes [174,175]. In summary, the architecture of gene regulatory networks influences how gene expression can evolve. A detailed understanding of tissue or stage specific gene regulatory networks and the integration of that knowledge into studies on the evolution of gene expression will certainly allow identifying general mechanisms generating variation in regulation. 

## 6. Context Dependency Should be Considered in Comparative Expression Studies

In the light of context dependent gene expression and gene regulation it is important to account for these aspects in comparative expression studies. Since high throughput sequencing methods are highly sensitive, not all identified genes in such studies may be directly associated with the trait of interest, but rather represent background noise. Few studies specifically tested whether complex tissue composition influences the sensitivity to detect gene expression differences. A RNAseq experiment in *D. melanogaster* compared genome wide gene expression in central nervous system tissue between wildtype and transgenic flies after RNA interference (RNAi) mediated cell-type specific downregulation of a ubiquitously expressed gene. Intriguingly, the authors could show that contamination by surrounding tissue was sufficient to hamper the identification of the artificially downregulated gene [176]. This specific example strongly suggests that restricting sequencing efforts to the tissue and time point of interest allows identifying differentially expressed genes as specific as possible. The same rationale applies for eQTL and ASE studies, since the analysis of complex organs composed of various cell types does not allow gaining cell type specific mechanistic insights (Table 2).

The lack of tissue specificity can partially be accounted for by cell type or tissue enrichment in model organisms that offer a versatile transgenic toolkit (see also Table 2). This approach usually requires the generation of transgenic individuals in which the target cell type or tissue is labeled by artificial fluorescent markers such as green fluorescent protein (GFP). Upon tissue dissociation, the labeled cells can be sorted by fluorescence-activated cell sorting (FACS) and classical bulk-RNAseq can be performed subsequently. This method has been successfully used to identify cell-type specific gene expression profiles [177,178,179] as well as to reveal candidate genes in evolutionary studies [180]. While this approach is restricted to genetically tractable model systems and requires in-depth information about the tissue of interest, recent advances in single cell RNA sequencing (scRNAseq) provide an excellent opportunity to gain cell type specific insights into gene expression of heterogeneous tissues without prior knowledge [124,125,126] (Table 2). A huge body of work has been published reporting for instance single cell atlases for various organisms such as embryos of *Drosophila melanogaster* [181], the cnidarian *Nematostella vectensis* [182], the planarian *Schmidtea mediterranea* [183] or the marine annelid *Platynereis dumerilii* [184]. Also, organ specific single cell atlases are being generated these days: In *Drosophila* for instance, new biological insights into the cell diversity, cell specific gene expression and gene regulation have been gained for entire aging brains [185], but also for parts of the brain such as the optic lobes [186] and the mid-brain [187]. The ability to reconstruct cell specific gene regulatory networks [188,189] from scRNAseq data provides the basis to relate comparative gene expression data to gene regulatory network architecture in a highly specific cellular context. Eventually, many high throughput sequencing applications to assess chromatin accessibility, histone modifications or transcription factor binding are applicable on single cell resolution (see Table 1 for details and references). Therefore, it is possible to compare the impact of genetic variation on these regulatory traits on single cell resolution. In summary, recent advances in single cell sequencing technologies provide an excellent opportunity to study context dependent gene expression in complex tissues. The combination of such methods with eQTL or ASE studies will allow revealing the impact of genetic variants on context dependent gene regulation.

**Figure 2:**
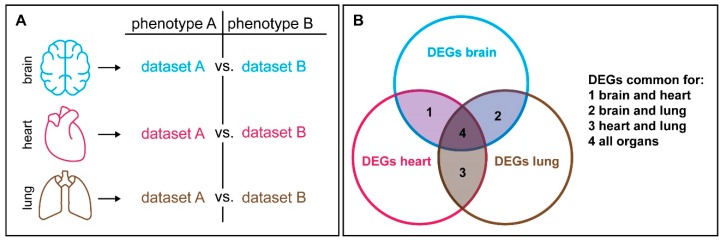
Generic factors that are expressed across different tissue can be excluded in correlation studies. (**A**) If specific candidate genes that are differentially expressed between phenotype A and B are supposed to be revealed, one can generate a comparable dataset for additional tissues. (**B**) Each pairwise comparison will reveal a certain number of differentially expressed genes (DEGs). The DEGs that are common in two (1–3) or all organs (4) are most likely generic factors that may be less informative for follow-up analyses.

Even if a tissue or stage of interest was selected as specific as possible, candidate gene lists obtained by comparative expression studies can further be specified by integration of prior knowledge about the molecular functions of differentially expressed genes. While many genes show context dependent expression, housekeeping genes, which fulfil generic tasks in each cell, are often stably expressed across different tissues. Comparative approaches can be used to exclude generic differentially expressed genes by analyzing which transcripts are consistently differentially expressed across different tissues or time points and can therefore be removed from candidate gene lists (Figure 2). It is also helpful to have some prior knowledge about molecular pathways and processes that are involved in regulating the phenotypic trait of interest. Variation in physiological traits may be associated with hormonal signals or enzymatic reactions, while morphological divergence is often linked to differences in underlying developmental processes. The growing Gene Ontology (GO) database coordinated by the Gene Ontology Consortium [190,191] provides an excellent basis for integrating differential gene expression and molecular functions. This tool allows to structure and categorize a list of candidate genes if no prior molecular or cellular knowledge for the trait of interest is available, by testing, whether a list of candidates is enriched in GO terms with a particular molecular or cellular function. Similarly, gene set enrichment analysis (GSEA) [192,193,194,195] can be employed to reveal if specific molecular or developmental pathways are involved in the development of the trait of interest [196]. Hence, the implementation of biological knowledge that recapitulates context specific information helps finding patterns in an otherwise unstructured dataset and helps to restrict the number of meaningful candidates.

## 7. Outlook

Although exciting insights into the evolution of gene expression and underlying regulatory mechanisms have been obtained, a few key questions remain to be answered. 

It has for instance rarely been tested, whether intra- and interspecific differences in gene expression provide any fitness advantage for the organisms. Since gene expression divergence is often associated with phenotypic variation, one potential approach to answer this question could be the thorough integration of gene expression data with phenotypic data of relevant traits. Such a combinatorial approach has been successfully applied to reveal candidate genes involved in different nest building behaviors among the two mouse species *Peromyscus polionotus* and *P. maniculatus*. QTL mapping revealed 498 candidate genes associated with behavioral differences. Tissue specific RNA sequencing, accounting for the fact that the studied behavior is controlled in a specific brain region, identified 23 differentially expressed genes within the QTL region, of which nine genes showed signatures of *cis*-regulatory divergence. One of these nine genes was subsequently functionally validated [32]. Similar combinatorial approaches have been applied to identify key candidate genes responsible for variation in salt tolerance in rice (*Oryza rufipogon*) [197] and flowering time in rape (*Brassica napus*) [198]. Population genetics data that provides genome wide insights into signatures of selection can equally be combined with comparative expression data to reveal meaningful candidate genes underlying divergence of relevant phenotypic traits [199]. The integration of tissue and stage specific comparative expression data with quantitative and population genetics approaches thus provides a powerful way to reveal those differentially expressed genes with a direct effect on relevant phenotypic traits. 

Another fundamental open question concerns the evolutionary forces underlying gene expression divergence. It will for instance be interesting to analyze the impact of genetic variants segregating in natural populations of species on various levels of the gene regulation machinery. Since gene regulation is highly context dependent, it will be important to study causal links between genetic variants and gene expression and gene regulation in as many different organisms, tissues, cell types and developmental stages. With functional genomics and transcriptomics methods being widely applicable (Table 1), we can now test, whether mechanistic insights obtained by highly coordinated consortia studying individual human cell lines as well as tractable genetic model systems such as yeast and *Drosophila* hold true in other study systems. 

## 8. Conclusions

Comparative genome-wide expression studies have been extensively used to reveal candidate factors to inform about the genotype–phenotype map (correlation studies) as well as to gain mechanistic insights into the evolution of gene regulation (eQTL and ASE). We argue that much more defined datasets must be generated in the future to fully account for the complexity and context dependency of gene regulation to increase the power to detect more meaningful candidate genes in correlation studies. We strongly believe that our current understanding of the evolution of gene expression provides a solid basis to incorporate new aspects of gene regulation, that are being revealed on a regular basis, to gain exciting new mechanistic insights into the evolutionary processes. There is still a sphere of cloudiness around the evolution of gene expression but digging deeper holds a chance of insight. 

## Figures and Tables

**Table 1:** Next generation sequencing techniques used for studying gene expression and gene regulation in evolutionary studies. Methods labelled with * require a reference genome.

Method	Key information
**RNAseq**	**Summary:** RNA is isolated and reverse transcribed into cDNA for library preparation and sequencing. **Practical considerations:** The most common protocol uses oligo-dT primers to enrich for polyadenylated RNAs for reverse transcription of processed mRNA [17] and the majority of lncRNAs [118]. Alternative protocols use total RNA and ribosome depletion prior to reverse transcription with random oligos to obtain other RNA molecules (e.g., immature mRNA, miRNA, and siRNA) [119]. For small RNA enrichment several commercial kits are available to select for molecule sizes less than 30 nucleotides [120]. **Applications:** Transcriptome generation for gene annotation including alternative isoforms (paired-end sequencing) and differential gene expression analysis between different samples (e.g., tissues, experimental conditions, populations of the same species or even species showing different phenotypes) [18,19,20,21,121]. RNAseq is also a useful tool for miRNA profiling and annotation [122] as well as differential expression of lncRNAs [123]. **Single cell application:** [124,125,126]
ATACseq*	**Summary:** Accessible chromatin regions which are not condensed by histones, are digested with a genetically modified transposase (Tn5). Nucleotide overhangs (tagmentation) are utilized for specific adapter ligation during the library preparation and sequencing [69,127]. This method substituted previous ones such as DNaseseq and FAIREseq, due to its simplicity and effectiveness. **Practical considerations:** Usually the protocol should be done with fresh tissue and a defined number of nuclei/cells (e.g 500–50,000 for mammalian tissues [127]) that have to be estimated prior to tagmentation. These technical aspects limit the number of samples that can be processed simultaneously. However, protocols were successfully applied to frozen tissue [128]. **Applications:** ATACseq is commonly used to complement RNAseq data to identify potential regulatory regions (enhancers) [129]. ATACseq can also be used to evaluate chromatin structure dynamics and epigenetic changes by providing information about histone position as well as a complementary approach to ChIPseq to characterize transcription factor and repressor (e.g., CTCF) occupancies [69]. **Single cell application:** [130,131]
ChIPseq*	**Summary:** DNA bound proteins (e.g., transcription factors, histones) are crosslinked and the chromatin is digested with restriction enzymes. Antibodies specific for the DNA-binding protein are used to isolate Protein-DNA fragments. After reversal of the crosslink and dissociation of the DNA short read sequencing libraries are prepared [132,133]. **Practical considerations:** This technique relies on previous knowledge about the DNA-binding proteins and available antibodies. **Applications:** ChIPseq is commonly used to generate genome wide data on protein-DNA interactions, mainly to determine transcription factor binding sites and their binding dynamics [134]. It has been used also to estimate histone modifications and nucleosome position between different species [72]. **Single cell application:** [135]
Hi-C*	**Summary:** DNA-binding proteins and chromatin are covalently crosslinked with formaldehyde and digested with a restriction enzyme. The resulting fragments are ligated to create chimeric molecules of DNA which are further isolated for library preparation and sequencing [136]. **Practical considerations:** Hi-C relies on restriction enzyme recognition sites which can create bias due to their heterogeneous distribution in the genome [137]. Alternative methods used DNase I [138] or micrococcus digestion [139] to overcome that issue. **Applications:** Hi-C is commonly used to identify global patterns of 3D genome conformation. Additionally, this method allows exploring how interactions between different chromosomal regions can affect gene regulation. The impact of chromatin topology on gene expression between species has been studied [64,66,140]. **Single cell application:** [141]
**BSseq **	**Summary:** DNA is treated with sodium bisulfite to deaminate cytosine bases into uracil (thymine after PCR) while methyl-cytosine bases are not affected [142]. The treated DNA is then digested for library preparation and sequencing [143]. **Practical considerations:** The deamination reaction usually has high yield, but small variations can create significant bias in the estimation of global methylation patterns [144]. Since cytosine is converted into thymine, the sequence complexity is reduced, and the strands are no longer complementary causing potential problems with the alignments. However, dedicated software has been developed to deal with the challenging BSseq data analysis (reviewed in [145]). **Applications:** This method is used to obtain genome wide patterns of DNA methylation which is an important epigenetic modification typically associated with gene expression repression [143]. In recent years, this method has been extensively applied to ecological and evolutionary studies [146,144]. **Single cell application:** [147,148]

**Table 2:** Comparison of different RNA sequencing methods.

	bulk-RNAseq of Whole Individuals	bulk-RNAseq with Prior Selection	scRNAseq
**What can I do?**			
Gain cell type specific gene expression	−	+/−	+
Identify overall gene expression profile	+	−	−
**What do I need?**			
Prior knowledge about the tissue or cells of interest	−	+	−
Transgenic organisms/fluorescently labeled cells	−	+	−
Specific technique to obtain tissue/cells	−	+/−	+
